# Supplementary material for: Latent classes of anthropometric growth in early childhood using uni- and multivariate approaches in a South African birth cohort
Source: PLoS One. 2025 Mar 25;20(3):e0319237. doi: 10.1371/journal.pone.0319237 (PMC11936193; doi:10.1371/journal.pone.0319237)
Supplement: S1 File — (PDF) [file pone.0319237.s001.pdf]

S1 File: Extended Methods

1.1 Study design

See figure S1 and tables S1-S2 for details on study design and cohort characteristics.

2.1 Study Outcomes Details

Growth measurements included Height and Weight from which standardised Body Mass Index (zBMI) and standardised Weight for Height (zWFH) were calculated. Height (recumbent length (recorded in centimetres) measured as distance from crown to foot using a Seca length-measuring mat (Seca; Hamburg, Germany) from birth until 24 months, after which this was recorded as standing height using a wall-mounted stadiometer (Panamed; Philippines) and weight (the mass of a subject (to the nearest 10 g) in light or no clothing using a Tanita digital platform scale (TAN1584; IL, USA)) were measured by trained study staff. Each measurement was taken twice per visit per child to serve as technical replicates. Measures within 0.5cm and 0.1kgs of each other for height and weight respectively were acceptable and the first measurement was used. If measures were not within this acceptable range, a third measurement was taken and used. For comparability, standardised growth responses (with respect to Growth References) were analysed within this report; these are denoted using "zGrowthResponse" such as zHeight for standardised height and zWeight for standardised weight. Growth responses as well as the number of observations per visit are summarised in Table S1.

Fenton growth references were used to calculate standardised growth scores for prematurely born children (before 37 weeks' gestation); for full term infants WHO references adjusted for gestational age were used until two years (as is the convention), (10–12). Standardised height (zHeight; derived using WHO and Fenton reference ranges), standardised weight (zWeight; derived using WHO and Fenton reference ranges), standardised body mass index (zBMI; weight in kg divided by height in meters squared, standardised using WHO reference ranges) and standardised Weight for Length/ Height (zWFH; derived using WHO reference ranges) were calculated.

As is convention, lying-down length was measured up to two years after which standing height was recorded. It is also well documented that recordings of length often contain more measurement error as it is difficult to keep infants still and equally stretched out during each follow up visit. To minimise this staff were trained repeatedly in growth measurements, a standardised operating procedure was used, and all measurements were done in triplicate.

2.2 Extended Statistical Methods

The modelling of the respective growth measures involved two overarching approaches: 1) Univariate- and 2) Multivariate Latent Class Mixed Models. Within the univariate modelling section each of the growth measures, zHeight, zWeight, zBMI and zWFH, was described and analysed independently. The multivariate approach allowed the growth measures to be analysed together, thus identifying groups of subjects that follow similar trajectories while considering more than one growth response. Using this multivariate approach, latent classes from a model based on zHeight and zWeight was considered as

an alternative to latent classes from the individual models for the calculated composite scores, zBMI and zWFH.

The final form of the mixed effect model for each of the responses takes the following structure:

$$y_{ij} = \beta_0 + \beta_1 x_{1ij} + b_1(x_{1ij} - l_1)_+ + b_2(x_{1ij} - l_2)_+ + \cdots + b_n(x_{1ij} - l_n)_+ + \mu_i + \epsilon_{ij}$$

where,

y_{ij} refers to the growth response measured for individual i at time point j,

x_{1ij} refers to the age of individual i at time point j,

μ_i captures the *subject specific random effect,*

ϵ_{ij} captures the *within-subject error,*

$l_{1\ to\ n}$ refer to knot locations with n included knots.

The linear mixed model assumes all subjects were sampled from the same population and thus can be represented using the same population-level relationship between the response and covariates. The latent class mixed model (LCMM) however assumes that the subjects have been sampled from K groups (or populations) and follow different latent profiles (14).

The LCMM method makes use of three models: a linear mixed effect model – the structural model (1), a measurement model – which relates the latent process to the observations (2) and a multinomial logistic model - which describes class membership (3).

(1) the structural model,

$$\Lambda_i(t)|_{c_i=k} = X_{L_1i}(t)^T\beta + X_{L_2i}(t)^T\nu_k + Z_i(t)^T u_{ik};\text{ where}$$

$\Lambda_i(t)$ refers to the latent process for subject i at timepoint t,

$c_i = k$ if subject belongs to latent class k,

and assume population composed of K underlying latent classes characterised by K, mean profiles of trajectories with latent class variable,

β describes the effects of the population level covariates, X_{L_1i},

within this study this refers to the time component and hence $X_{L_1i}(t)^T\beta$ refers to the piecewise-linear spline specification previously described,

ν_k describes the effects of group level covariates, X_{L_2i},

u_{ik} describes the subject specific profiles within this model,

thus, capturing the repeated measures nature of this data. which describes the growth responses over time and allows for repeated measures data.

(2) the measurement model,

$$Y_{ij} = H\left(\Lambda_i\left(t_{ij}\right) + \epsilon_{ij};\eta\right) \quad \text{where}$$

Y_{ij} denotes the response for subject i at occasion j,

H is a parameterized (with η) link function,

ϵ_{ij} captures within-subject error,

allowing for different types of response variables and a nonlinear relationship, which relates the latent process to the observed responses.

(3) the multinomial logistic model,

$$\pi_{ik} = P(c_i = k|X_{ci}) = \frac{e^{\xi_{0k} + X_{ci}^T \xi_{1k}}}{\sum_{l=1}^{K} e^{\xi_{0l} + X_{ci}^T \xi_{1l}}}$$

where:

X_{ci} refers to covariates that are included as control variables within the class allocation process,

ξ_{0k} is the intercept for class k,

ξ_{1k} is the vector of class-specific parameters associated with the time-independent covariates X_{ci},

describing group membership, based on a multinomial logistic model from which subjects are allocated to the class with the greatest resulting probability (14).

Within this study we did not consider the effect of additional covariates and hence this is simplified to the following:

(1) the structural model,

$$\Lambda_i(t)|_{c_i=k} = X_{L_1 i}(t)^T \beta + Z_i(t)^T u_{ik};$$

(2) the measurement model,

$$Y_{ij} = H\left(\Lambda_i(t_{ij}) + \epsilon_{ij}; \eta\right)$$

(3) the multinomial logistic model,

$$\pi_{ik} = P(c_i = k|X_{ci}) = \frac{e^{\xi_{0k}}}{\sum_{l=1}^{K} e^{\xi_{0l}}}$$

The LCMM model can be extended to model multiple responses simultaneously by fitting multivariate mixed models and multivariate latent class mixed models for multivariate longitudinal response variables. In this instance, the models previously described are

extended to allow observations from *m* responses, from i individuals at j timepoints. This is described in greater detail within Figure S2.

References

1. World Health Organization. WHO child growth standards : length/height-for-age, weight-for-age, weight-for-length, weight -for-height and body mass index-for-age : methods and development [Internet]. World Health Organization; 2006 [cited 2023 Aug 2]. Available from: https://apps.who.int/iris/handle/10665/43413

2. Fenton TR, Kim JH. A systematic review and meta-analysis to revise the Fenton growth chart for preterm infants. BMC Pediatr. 2013 Apr 20;13(1):59.

3. Fenton TR, Nasser R, Eliasziw M, Kim JH, Bilan D, Sauve R. Validating the weight gain of preterm infants between the reference growth curve of the fetus and the term infant. BMC Pediatr. 2013 Jun 11;13(1):92.

4. Proust-Lima C, Philipps V, Liquet B. Estimation of Extended Mixed Models Using Latent Classes and Latent Processes: The R Package lcmm. J Stat Softw. 2017 Jun 1;78:1–56.
