# Supplementary material for: Latent classes of anthropometric growth in early childhood using uni- and multivariate approaches in a South African birth cohort
Source: PLoS One. 2025 Mar 25;20(3):e0319237. doi: 10.1371/journal.pone.0319237 (PMC11936193; doi:10.1371/journal.pone.0319237)
Supplement: S2 File — (PDF) [file pone.0319237.s002.pdf]

S2: Latent Growth Response Analysis Results

3.1: Longitudinal Growth Responses

Describing Growth Responses over Time:

The zHeight and zWeight, zBMI and zWFH standardized measurements over time were modelled using mixed effect models with piecewise linear splines to capture the time component. Between 3 and 4 knots were placed between ages 0.25 and 2.5 years for the different growth measures and the resulting mean profiles are illustrated in the supplementary materials (Table S3 and Fig S4). The early placement of the knots with no knots beyond age 2.5 reflects that most of the changes in trajectory structure occurred before two years of age. These changes in trajectory structure may also be a feature of more frequent data collection during the infancy. To determine whether the choice of knot location impacted the subsequently identified latent profiles, the longitudinal profiles given additional knots placed at later time points (ages 0.25,0.75,1,1.5,2,3,4; Fig S5) were compared to those described in Figure S4. The profiles did not show clear deviation, and thus the knot locations specified in Table S3 were used.

3.3: Stability Testing - Selecting K, the number of latent classes:

With the previously described broken stick specifications of the longitudinal process, latent class mixed models were fit to each of the respective growth measures, zHeight, zWeight, zBMI and zWFH, individually. Additionally, this approach was also applied to the multivariate response, zHeight + zWeight. An important step within this process was choosing the appropriate number of latent classes, k.

Figure S6 illustrates the process whereby the number of latent classes (k) for the trajectories of zHeight was selected. Figure S6(A) shows the fit statistics for 1 to 5 latent classes. In this case the more classes, the lower the BIC. Similarly, the entropy for k=5 was the greatest (Fig S6(A)). The class sizes when five latent classes were considered were still greater than 5%, however the Figure S6(D) shows a lack of stability when k=5. As a compromise, k=4 was chosen as the optimal class number as the fit statistics were very similar to that of k=5, while the stability shown in Figure S6(C) is consistent.

This process was repeated for all growth responses, Figures S7-S11 in the supplementary materials. For zWeight, four latent classes were identified as optimal (Fig S7). Three latent classes were identified as optimal within zBMI and zWFH (Fig S8-S9). When considering the multivariate zHeight+zWeight model, five latent classes were identified as optimal (Fig S10-S11).

3.7: Link Function

When considering the link functions of this multivariate model that connects the observed process to the latent process, the slope of the zWeight link covers a greater range of the longitudinal process, thus the influence of weight may thus also be greater in predicting class allocations (Fig S20). A cubic function with three equally spaced knots was also considered as a more appropriate link function, while the fit characteristics produced were slightly better, the final profiles identified were almost identical (Fig S21) and thus the less complex linear link was used.

3.8: Comparison of Latent Class Allocations:

The correspondence between latent class allocations for individual children based on different growth measurements and on allocations based on univariate versus multivariate models are shown in Tables S10-12. Table 1 illustrates the correspondence between allocations based on zHeight, zWeight and zHeight and zWeight within a joint model in a three-way contingency table. In contrast, Tables S10-S11 summarise the agreement between composite measures of height and weight (zBMI and zWFH) and the class allocations based on models for joint zWeight and zHeight. Finally, Table S12 summarises the agreement between the latent classes identified given zWFH or zBMI.

Comparison of class allocations based on composite measures (zWFH and zBMI) to that based on joint zHeight and zWeight measurements:

Sixty Four percent of children were allocated to the “Gradual Increase to Slightly Above Expected” zWFH class, while based on the joint zHeight + zWeight response a large proportion of these children were allocated to all five classes (Table S10); this indicates that the zHeight + zWeight approach is able to separate children into growth classes that would have otherwise been lost if only zWFH was used as input. Interestingly, 49.5 percent of individuals allocated to zHeight+zWeight class (i) were allocated to the zWFH “Gradual Decrease to Expected” class, which is substantially higher than the overall percentage, 29%, of individuals allocated to this class, thus showing a strong agreement between these profiles. However, looking at the descriptions of these trajectories, the multivariate profiles are described as a “Sharp Decrease to Low” within zHeight and

“Gradual Decrease to Low” within zWeight while the zWFH trajectory does not suggest low zWFH. Thus, while an individual may be identified with abnormal growth through zWeight or zHeight, the use of zWFH may miss such a diagnosis.

In Table S11, the “Slight Increase, Slight Decrease to Expected” zBMI class shows a strong association with the multivariate class (i) where low zHeight and zWeight was observed. Additionally, the “Gradual Increase to High” class shows a strong association with multivariate classes (iii) and (v) which illustrate very different zHeight and zWeight profiles. Multivariate class (iii) describes children with below expected zHeight and expected zWeight scores, while multivariate class (v) illustrates children with expected zHeight and above expected zWeight scores. Thus, while subjects may have a similar relationship between zHeight and zWeight leading to allocation of the same zBMI class, the multivariate zHeight+zWeight approach is able distinguish between these individuals.

Finally, the comparison of agreement between latent trajectories identified when using zWFH and zBMI is presented (Table S12). The strongest associations exist between the zWFH “Sharp Increase to Above Expected” with the zBMI “Gradual Increase to High” classes, the zWFH “Gradual Decrease to Expected” and zBMI “Slight Increase, Slight Decrease to Expected” classes and the zWFH “Gradual Increase to Slightly High” and zBMI “Sharp Increase to High” classes. These associated classes show similar levels at which they settle, however the rate of change up until this point is varied between classes identified using zWFH and zBMI. While the allocation of classes was not entirely in agreement given these two responses, this does suggest that given either zWFH or zBMI

as input, the latent trajectories identified across responses may represent similar growth profiles.
