# Supplementary figures and images for: Latent classes of anthropometric growth in early childhood using uni- and multivariate approaches in a South African birth cohort
Source: PLoS One. 2025 Mar 25;20(3):e0319237. doi: 10.1371/journal.pone.0319237 (PMC11936193; doi:10.1371/journal.pone.0319237)

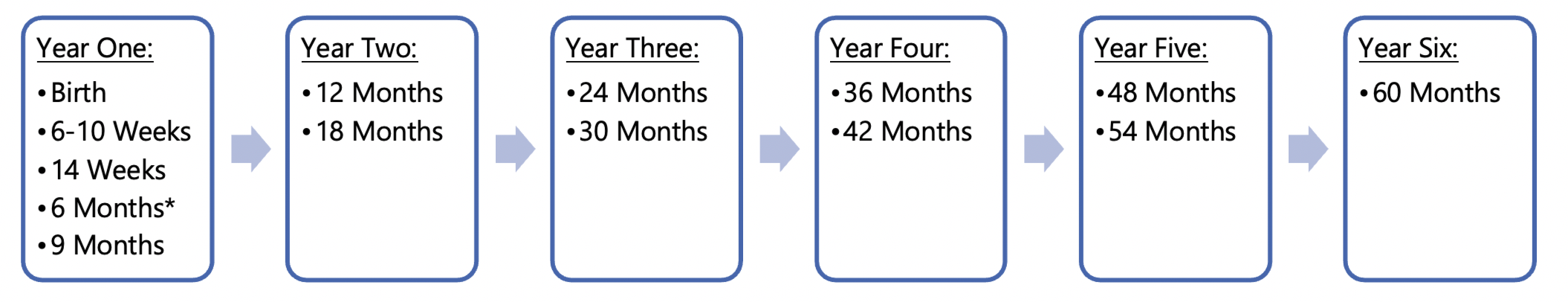

Supplement: S1 Fig — (TIF) [file pone.0319237.s003.tif]

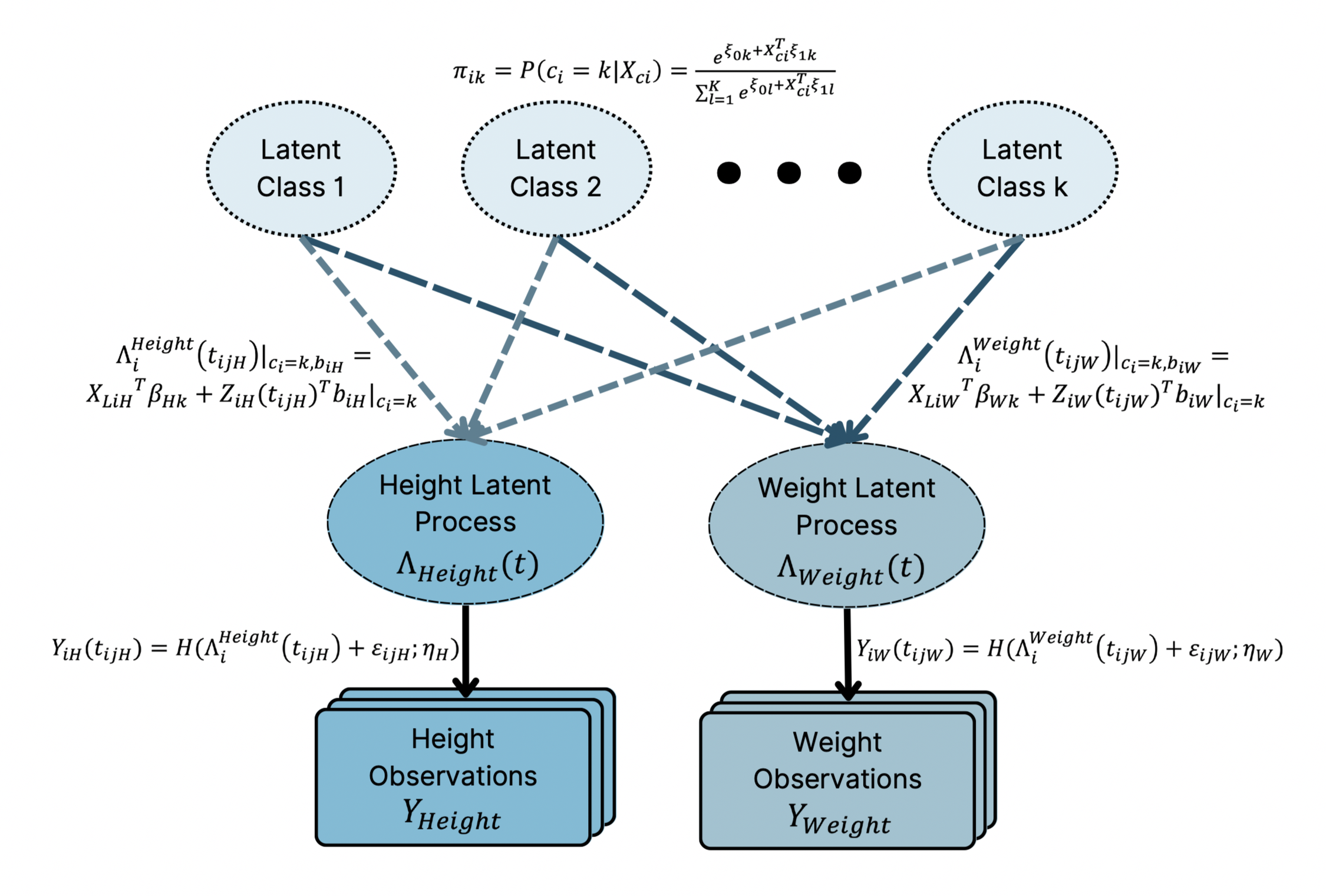

Supplement: S2 Fig — Diagram illustrating the structure of the multivariate LCMM process when considering distinct latent structures for zHeight and zWeight responses respectively. Additional detail as well as specification of terms can be found within Appendix B in the supplementary materials. (TIF) [file pone.0319237.s004.tif]

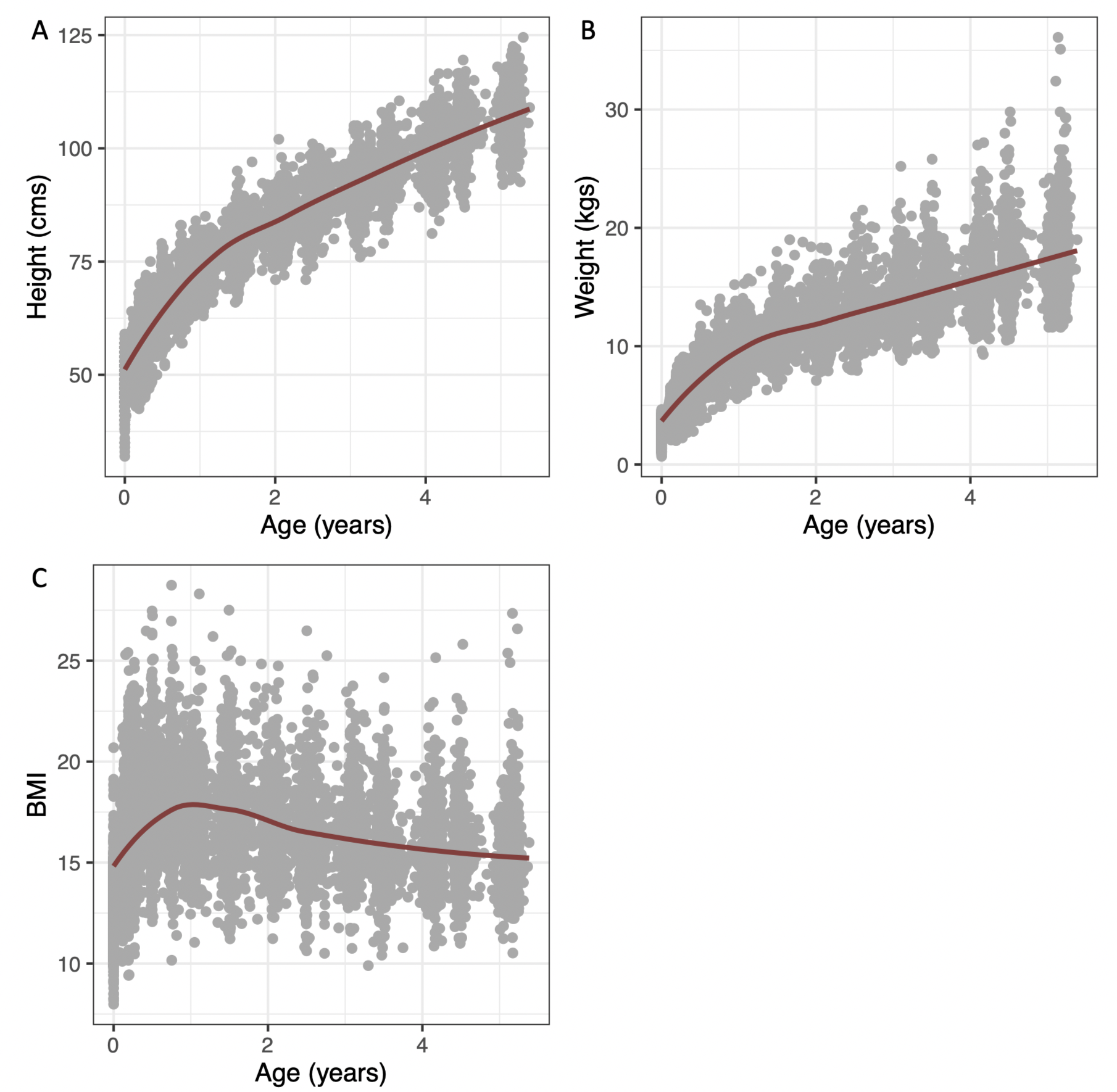

Supplement: S3 Fig — Observed growth measurements over time with a smoothed average indicated with a burgundy line for A) Height, B) Weight and C) BMI. (TIF) [file pone.0319237.s005.tif]

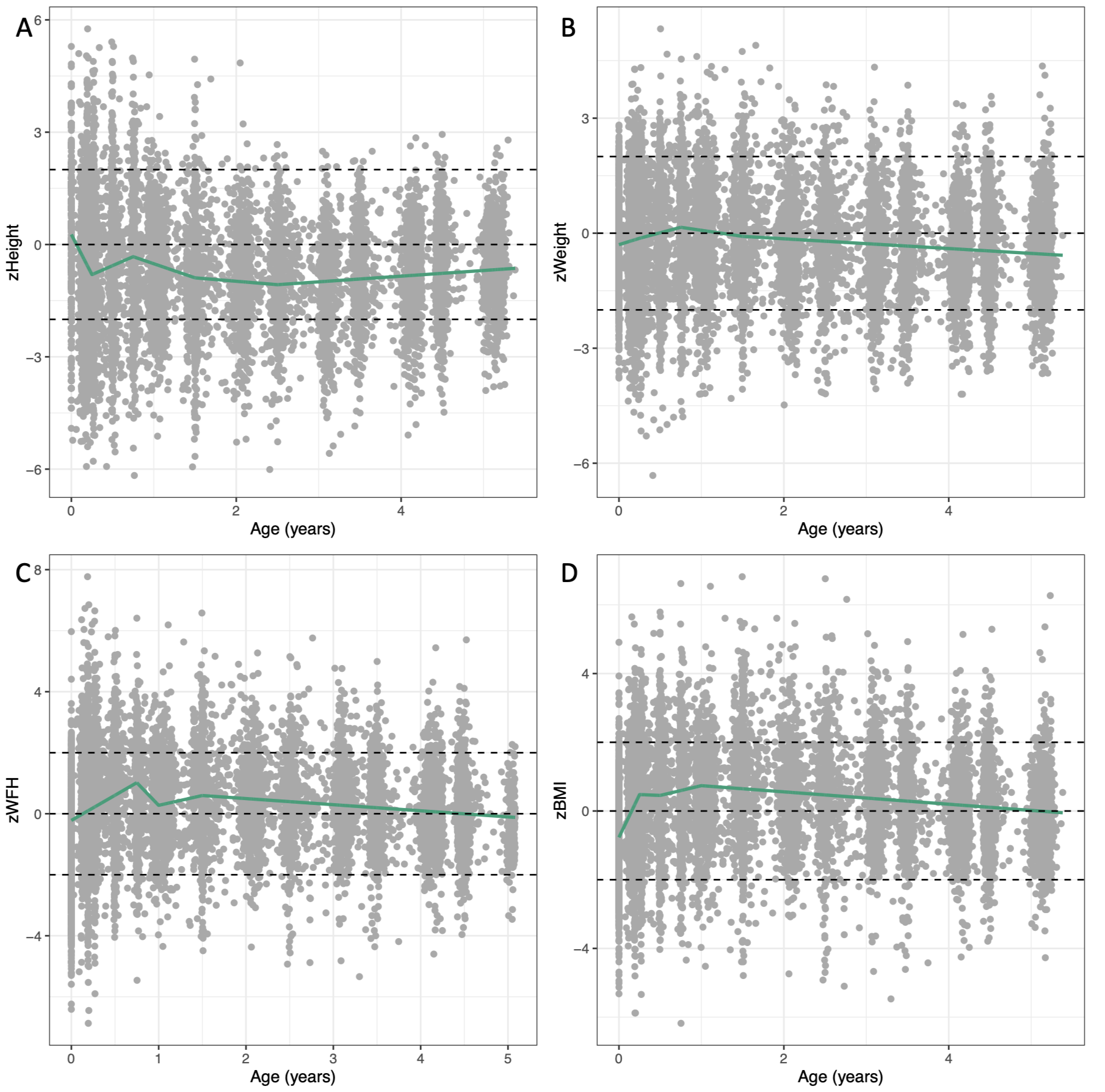

Supplement: S4 Fig — Standardised growth measurements over time with the average trajectory described through a piecewise-linear spline indicated with a green line for A) zHeight, B) zWeight, C) zWFH and D) zBMI. (TIF) [file pone.0319237.s006.tif]

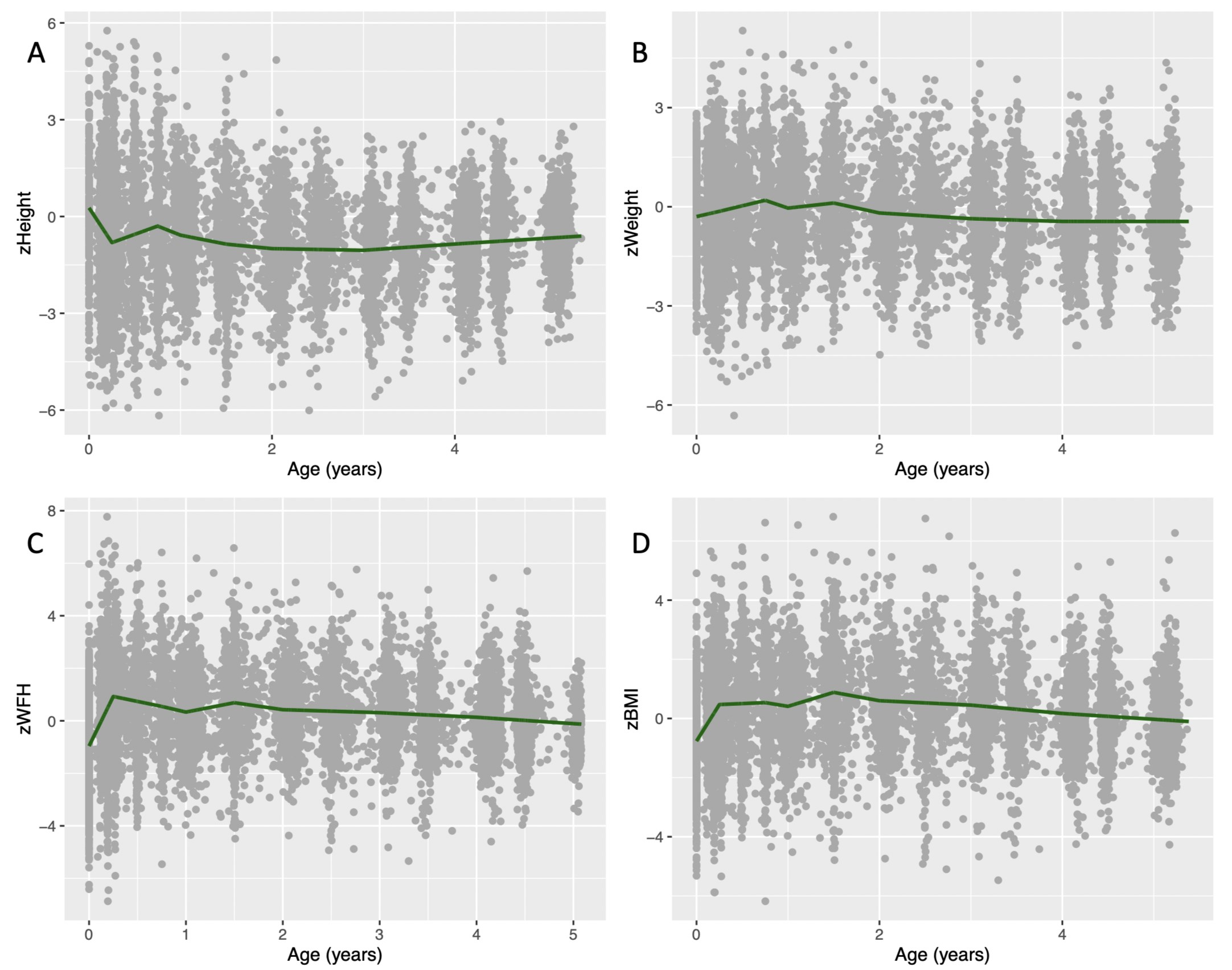

Supplement: S5 Fig — Standardised growth measurements over time with the average trajectory described through a piecewise-linear spline indicated with a green line for A) zHeight, B) zWeight, C) zWFH and D) zBMI given additional knots placed at timepoints (0.25,0.75,1,1.5,2,3,4). (TIF) [file pone.0319237.s007.tif]

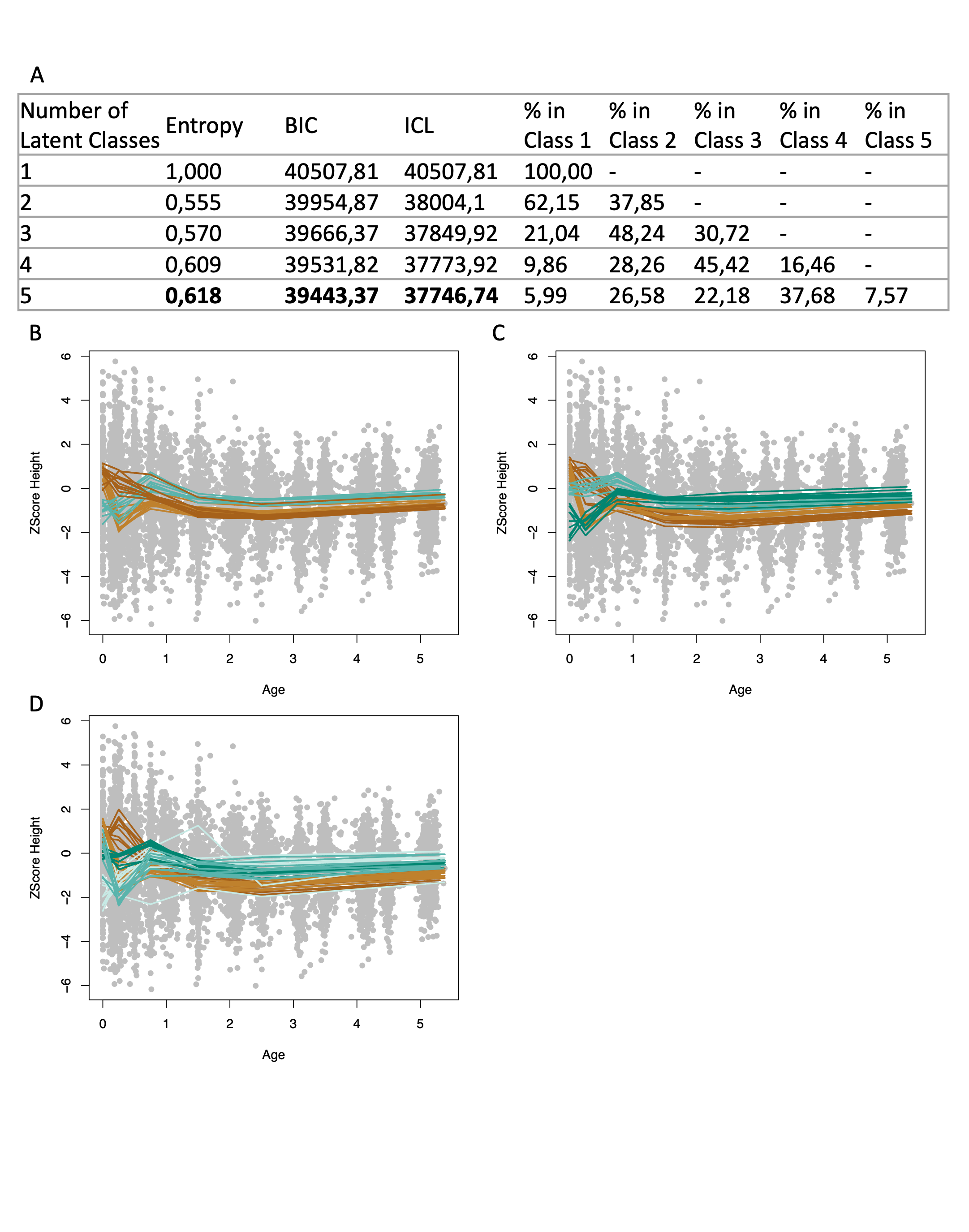

Supplement: S6 Fig — Information used to choose the appropriate value for k, the number of latent classes within standardised Height. A) Fit statistics for k = (1:5). Profiles of LCMM Classes identified within standardised Height using a randomly selected 50% of subjects, repeated 10 times for B) k = 3, C) k = 4 and D) k = 5. (TIF) [file pone.0319237.s008.tif]

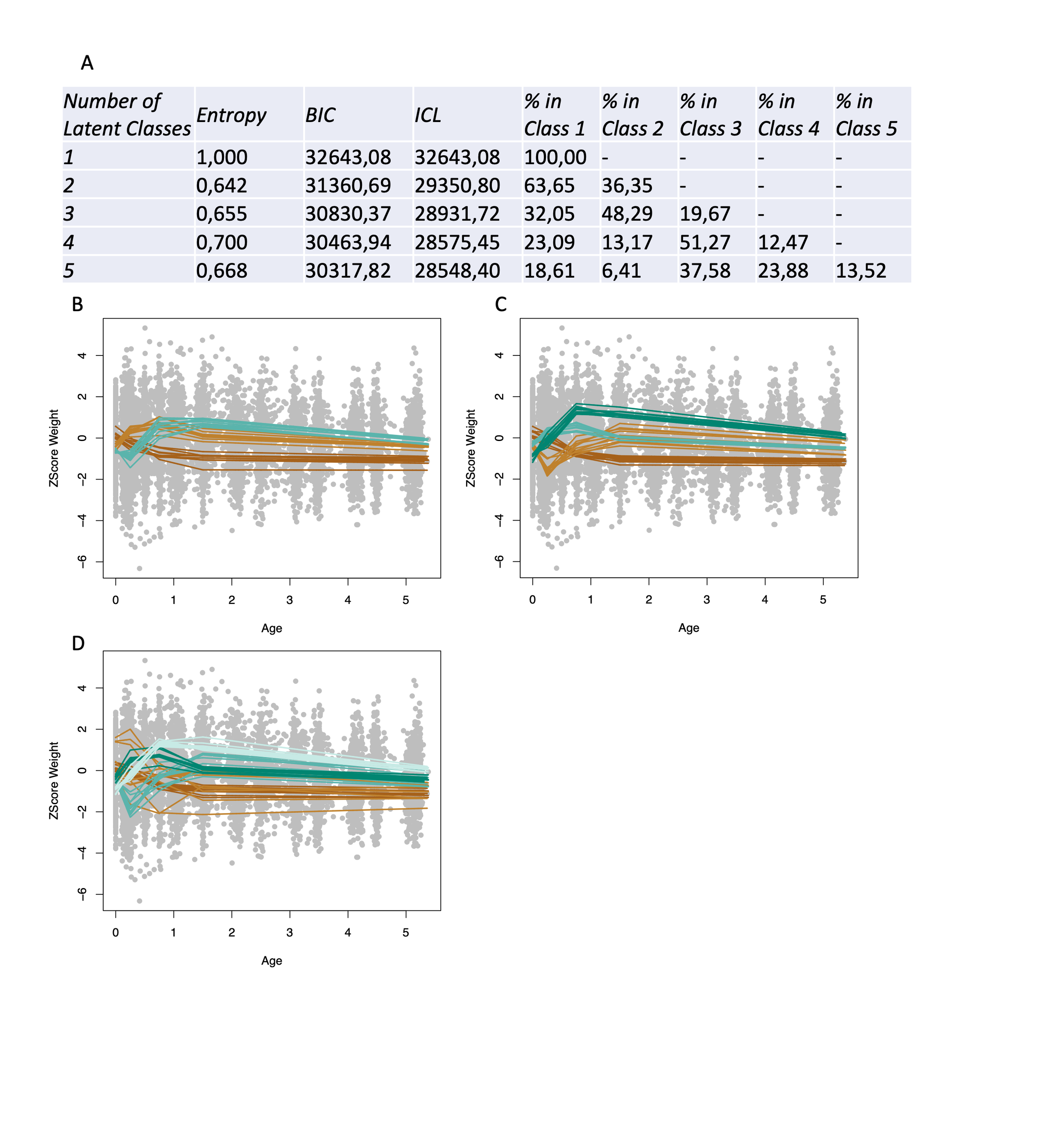

Supplement: S7 Fig — Information used to choose the appropriate value for k, the number of latent classes within standardised Weight. A) Fit statistics for k = (1:5). Profiles of LCMM Classes identified within standardised Weight using a randomly selected 50% of subjects, repeated 10 times for B) k = 3, C) k = 4 and D) k = 5. (TIF) [file pone.0319237.s009.tif]

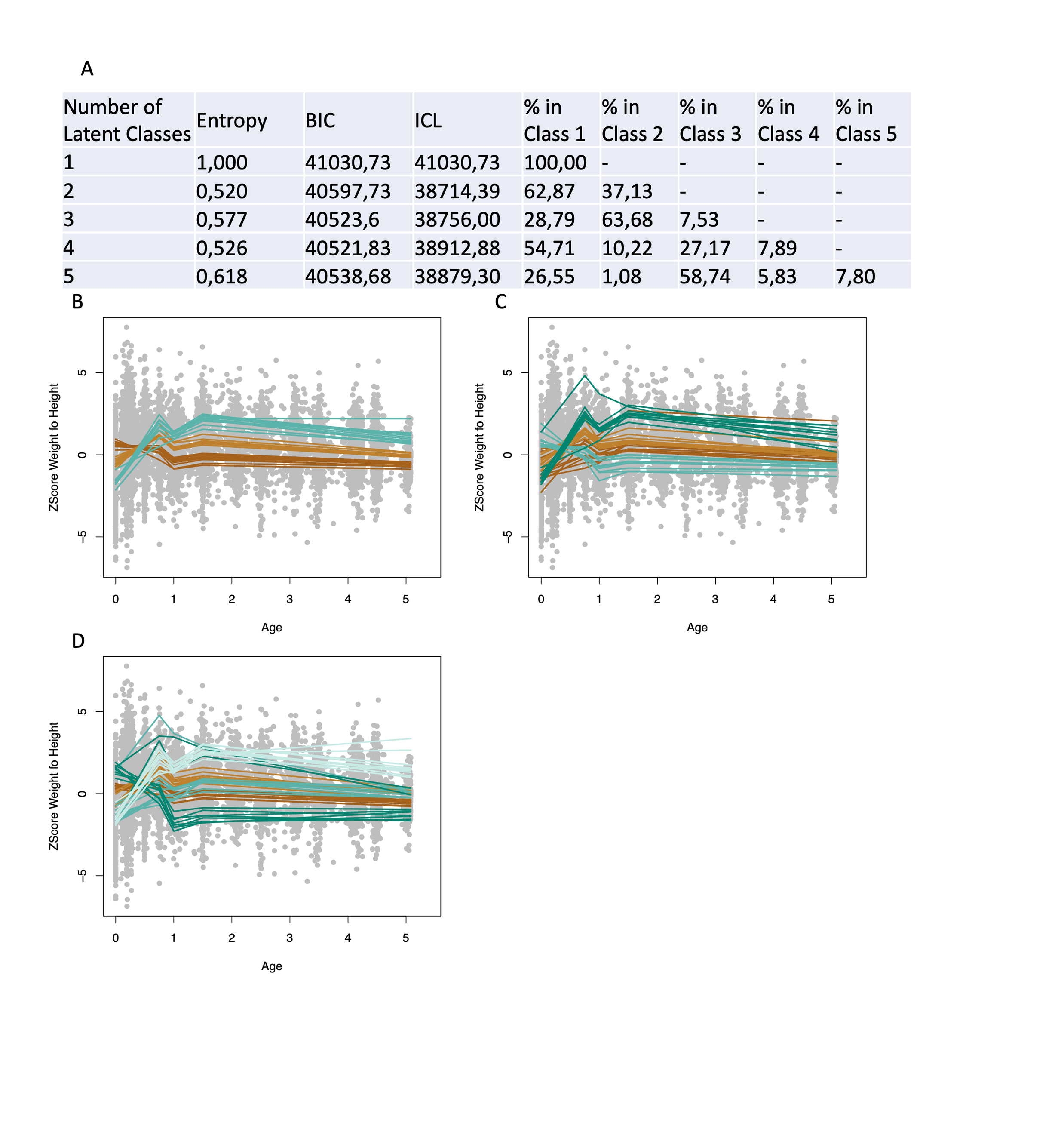

Supplement: S8 Fig — Information used to choose the appropriate value for k, the number of latent classes within standardised WFH A) Fit statistics for k = (1:5). Profiles of LCMM Classes identified within standardised WFH using a randomly selected 50% of subjects, repeated 10 times for B) k = 3, C) k = 4 and D) k = 5. (TIF) [file pone.0319237.s010.tif]

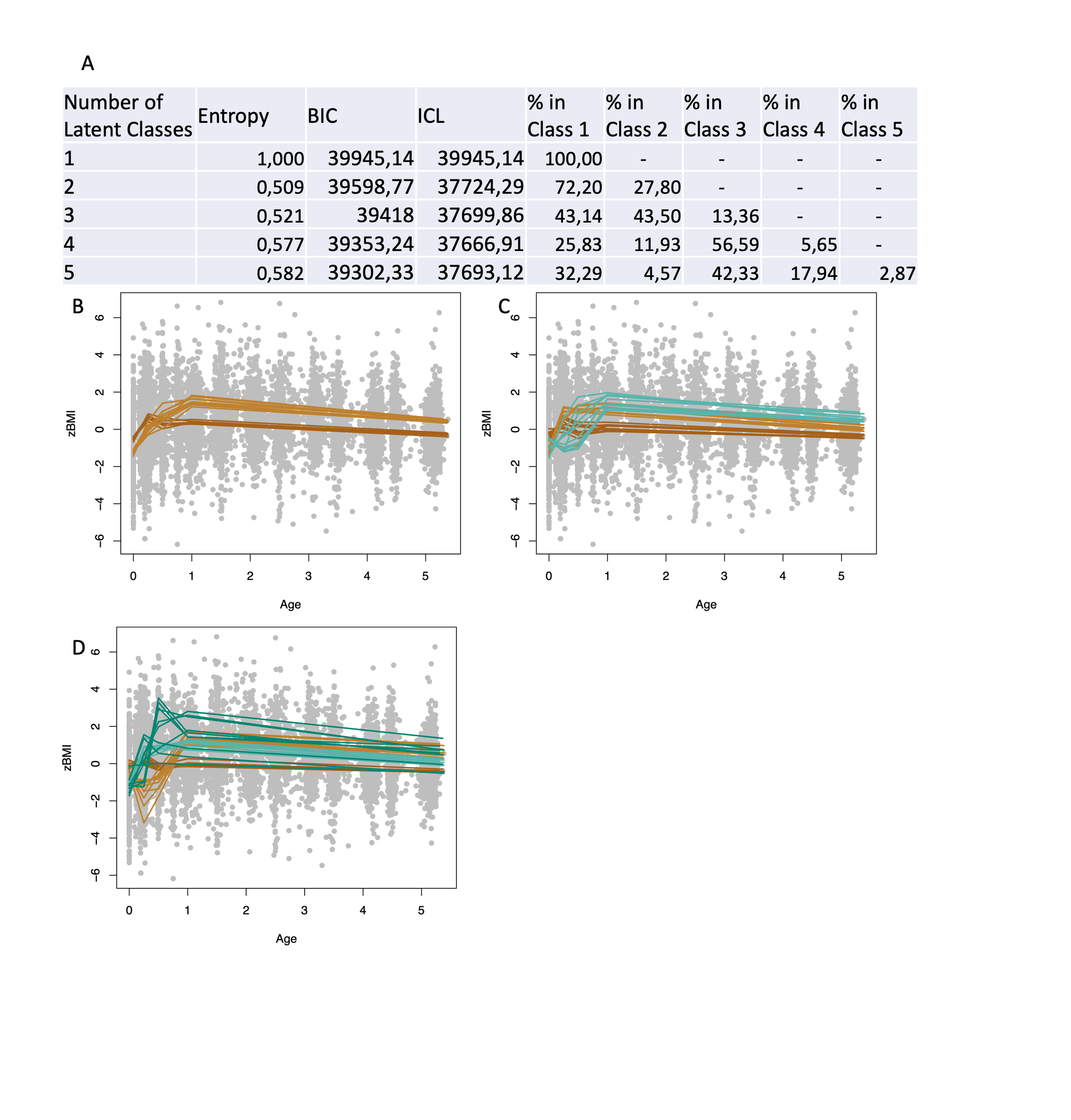

Supplement: S9 Fig — Information used to choose the appropriate value for k, the number of latent classes within standardised BMI A) Fit statistics for k = (1:5). Profiles of LCMM Classes identified within standardised BMI using a randomly selected 50% of subjects, repeated 10 times for B) k = 3, C) k = 4 and D) k = 5. (TIF) [file pone.0319237.s011.tif]

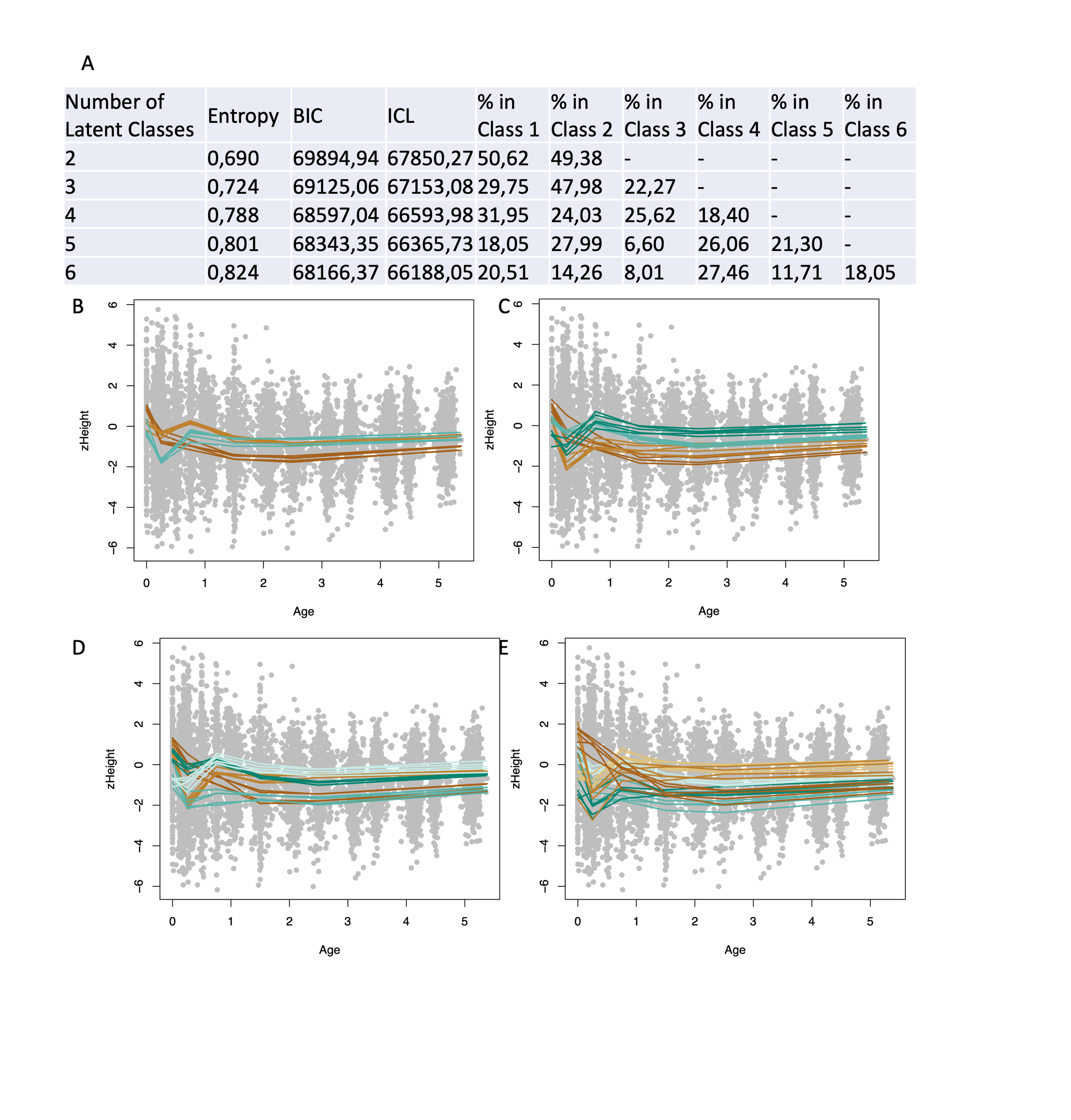

Supplement: S10 Fig — Information used to choose the appropriate value for k, the number of latent classes within zHeight + zWeight. Profiles of LCMM Classes identified within zHeight + zWeight using a randomly selected 50% of subjects, repeated 10 times for A) k = 3, B) k = 4, C) k = 5 and D) k = 6 illustrated using zHeight. (TIF) [file pone.0319237.s012.tif]

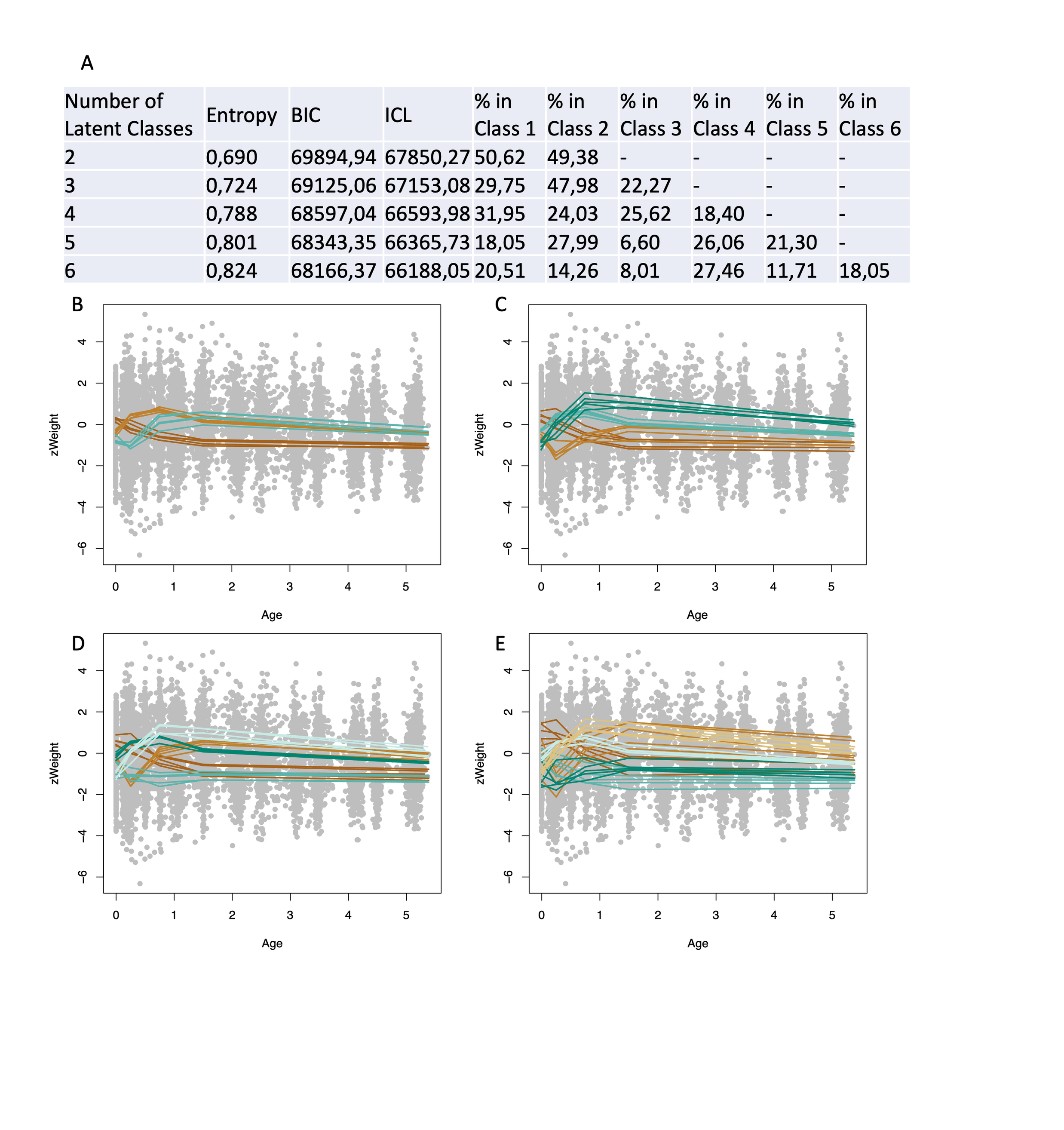

Supplement: S11 Fig — Information used to choose the appropriate value for k, the number of latent classes within zHeight + zWeight A) Fit statistics for k = (1:6). Profiles of LCMM Classes identified within zHeight + zWeight using a randomly selected 50% of subjects, repeated 10 times for B) k = 3, C) k = 4, D) k = 5 and E) k = 6, illustrated using zWeight. (TIF) [file pone.0319237.s013.tif]

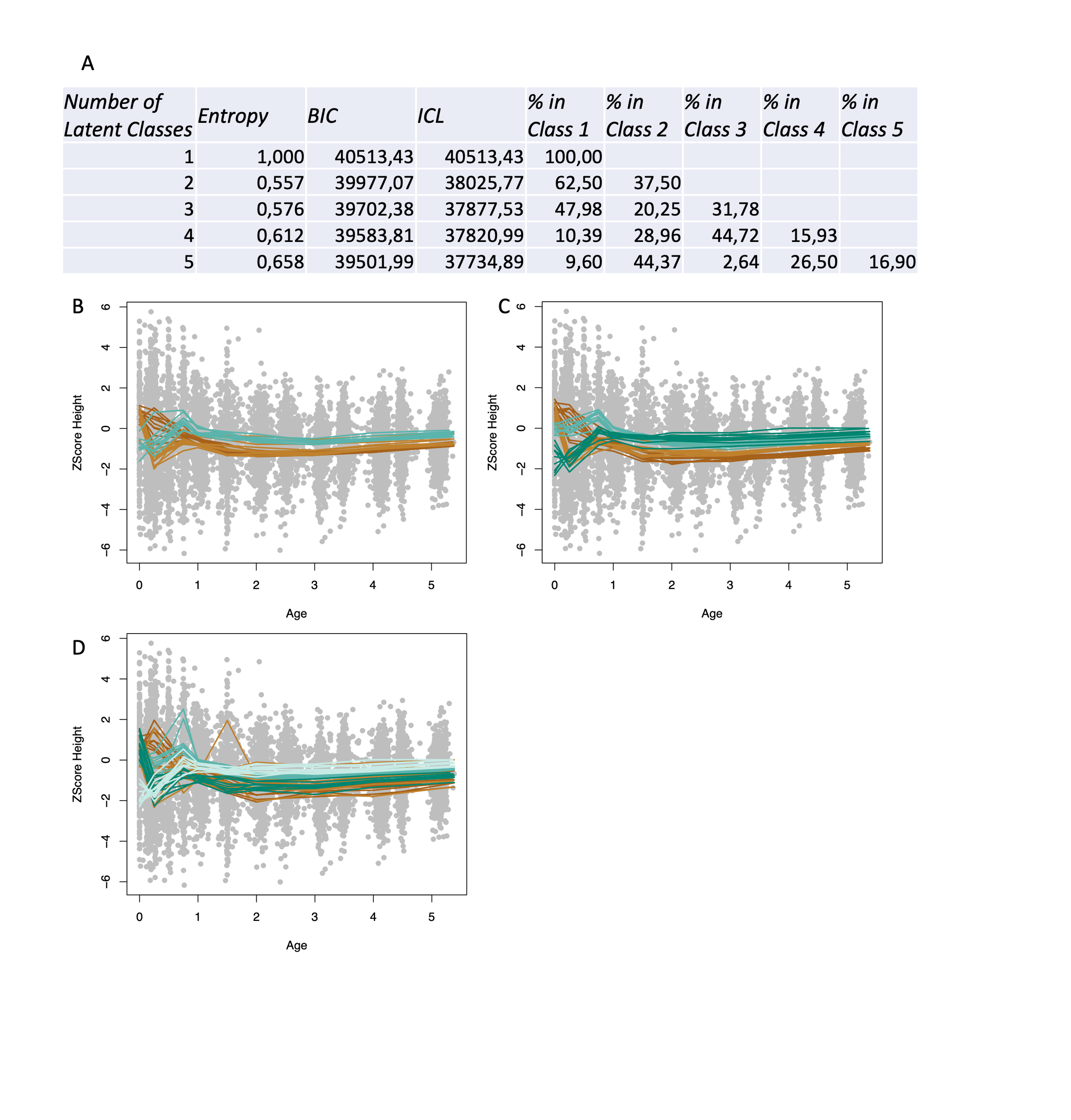

Supplement: S12 Fig — Information used to choose the appropriate value for k, the number of latent classes within standardised Height, given a piecewise linear spline model specification with knots places at (0.25,0.75,1,1.5,2,3,4). A) Fit statistics for k = (1:5). Profiles of LCMM Classes identified within standardised Height using a randomly selected 50% of subjects, repeated 10 times for B) k = 3, C) k = 4 and D) k = 5. (TIF) [file pone.0319237.s014.tif]

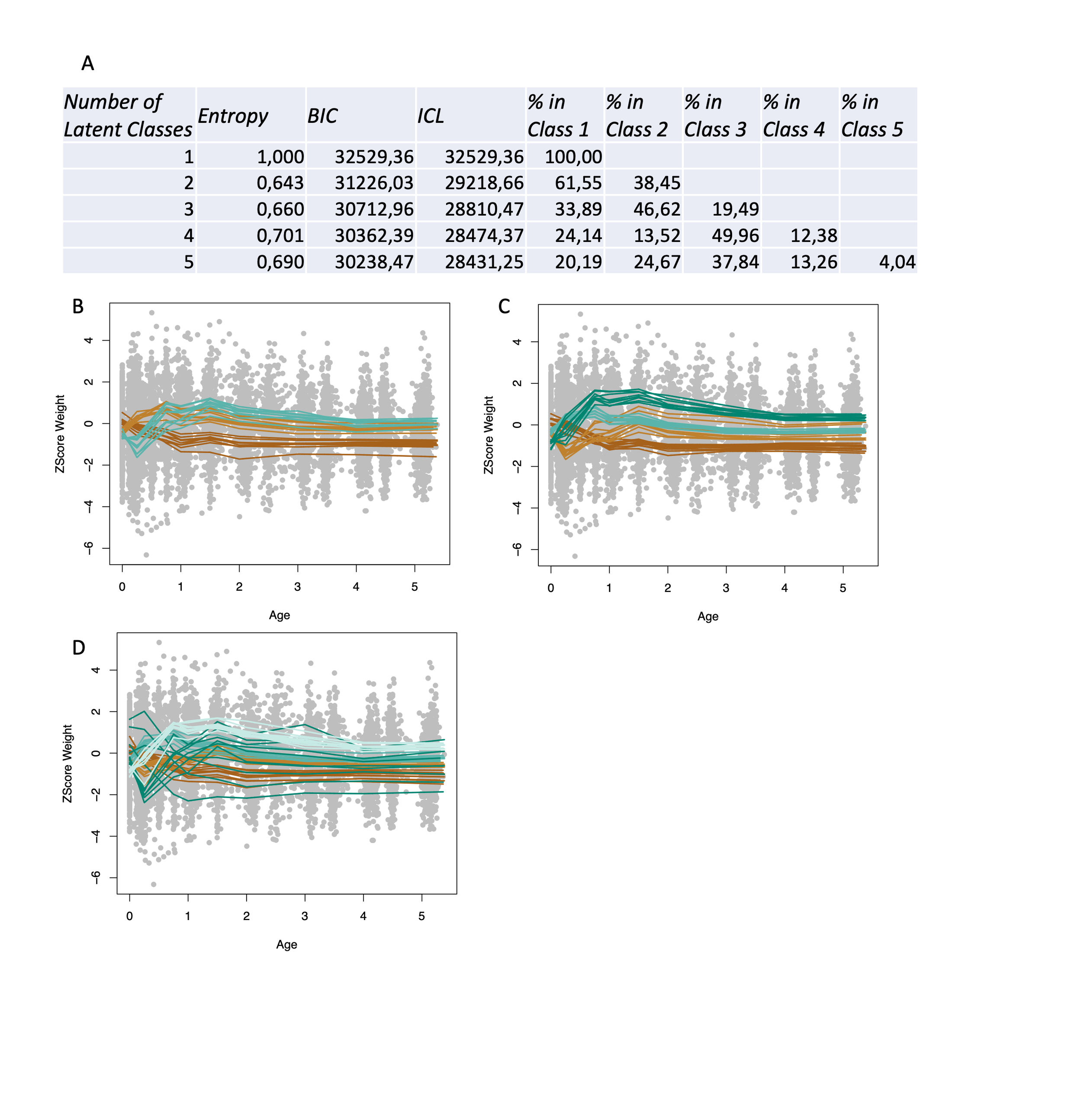

Supplement: S13 Fig — Information used to choose the appropriate value for k, the number of latent classes within standardised Weight, given a piecewise linear spline model specification with knots places at (0.25,0.75,1,1.5,2,3,4). A) Fit statistics for k = (1:5). Profiles of LCMM Classes identified within standardised Weight using a randomly selected 50% of subjects, repeated 10 times for B) k = 3, C) k = 4 and D) k = 5. (TIF) [file pone.0319237.s015.tif]

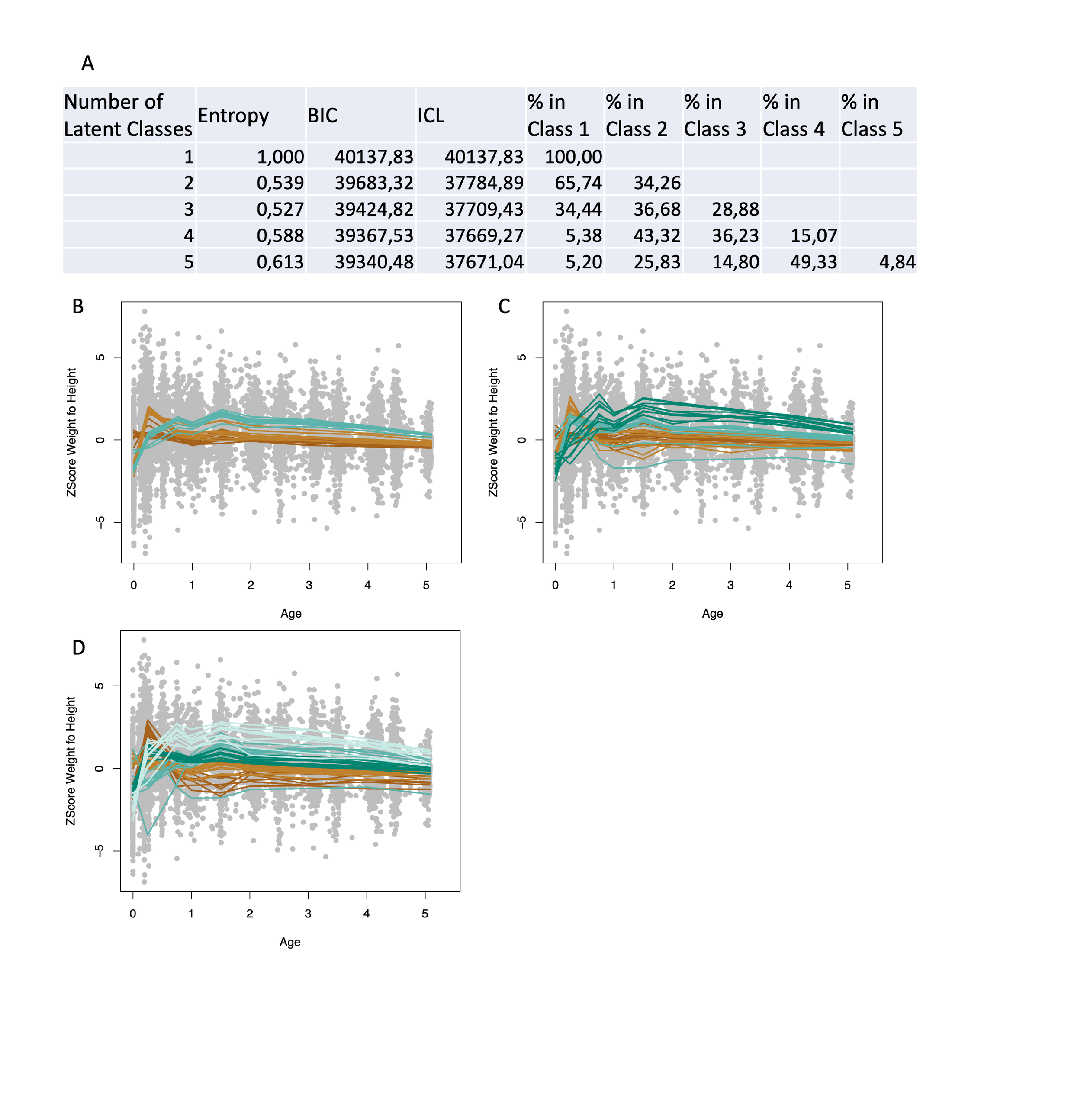

Supplement: S14 Fig — Information used to choose the appropriate value for k, the number of latent classes within standardised WFH, given a piecewise linear spline model specification with knots places at (0.25,0.75,1,1.5,2,3,4). A) Fit statistics for k = (1:5). Profiles of LCMM Classes identified within standardised WFH using a randomly selected 50% of subjects, repeated 10 times for B) k = 3, C) k = 4 and D) k = 5. (TIF) [file pone.0319237.s016.tif]

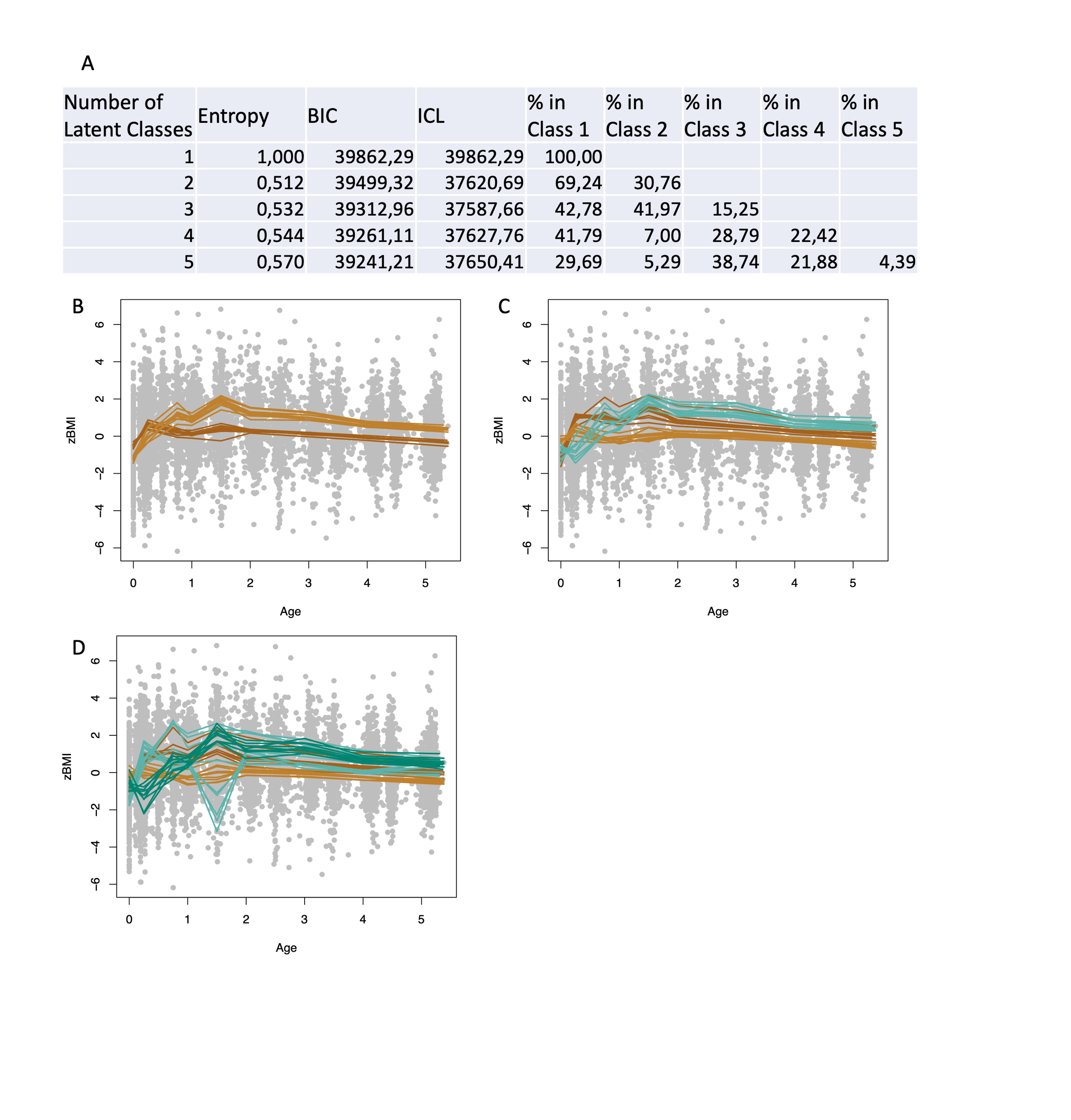

Supplement: S15 Fig — Information used to choose the appropriate value for k, the number of latent classes within standardised BMI, given a piecewise linear spline model specification with knots places at (0.25,0.75,1,1.5,2,3,4). A) Fit statistics for k = (1:5). Profiles of LCMM Classes identified within standardised BMI using a randomly selected 50% of subjects, repeated 10 times for B) k = 3, C) k = 4 and D) k = 5. (TIF) [file pone.0319237.s017.tif]

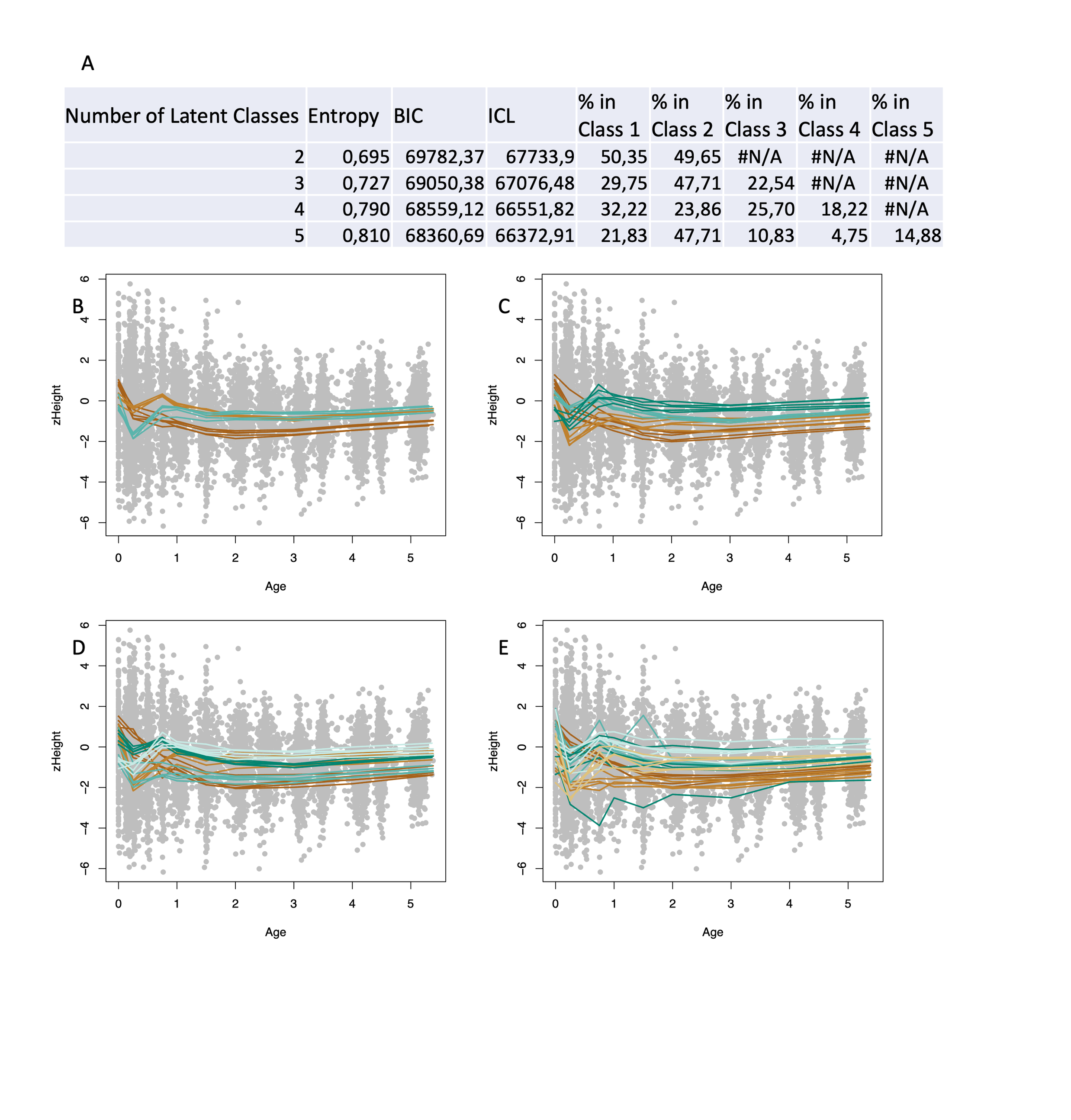

Supplement: S16 Fig — Information used to choose the appropriate value for k, the number of latent classes within zHeight + zWeight, given a piecewise linear spline model specification with knots places at (0.25,0.75,1,1.5,2,3,4). Profiles of LCMM Classes identified within zHeight + zWeight using a randomly selected 50% of subjects, repeated 10 times for A) k = 3, B) k = 4, C) k = 5 and D) k = 6 illustrated using zHeight. (TIF) [file pone.0319237.s018.tif]

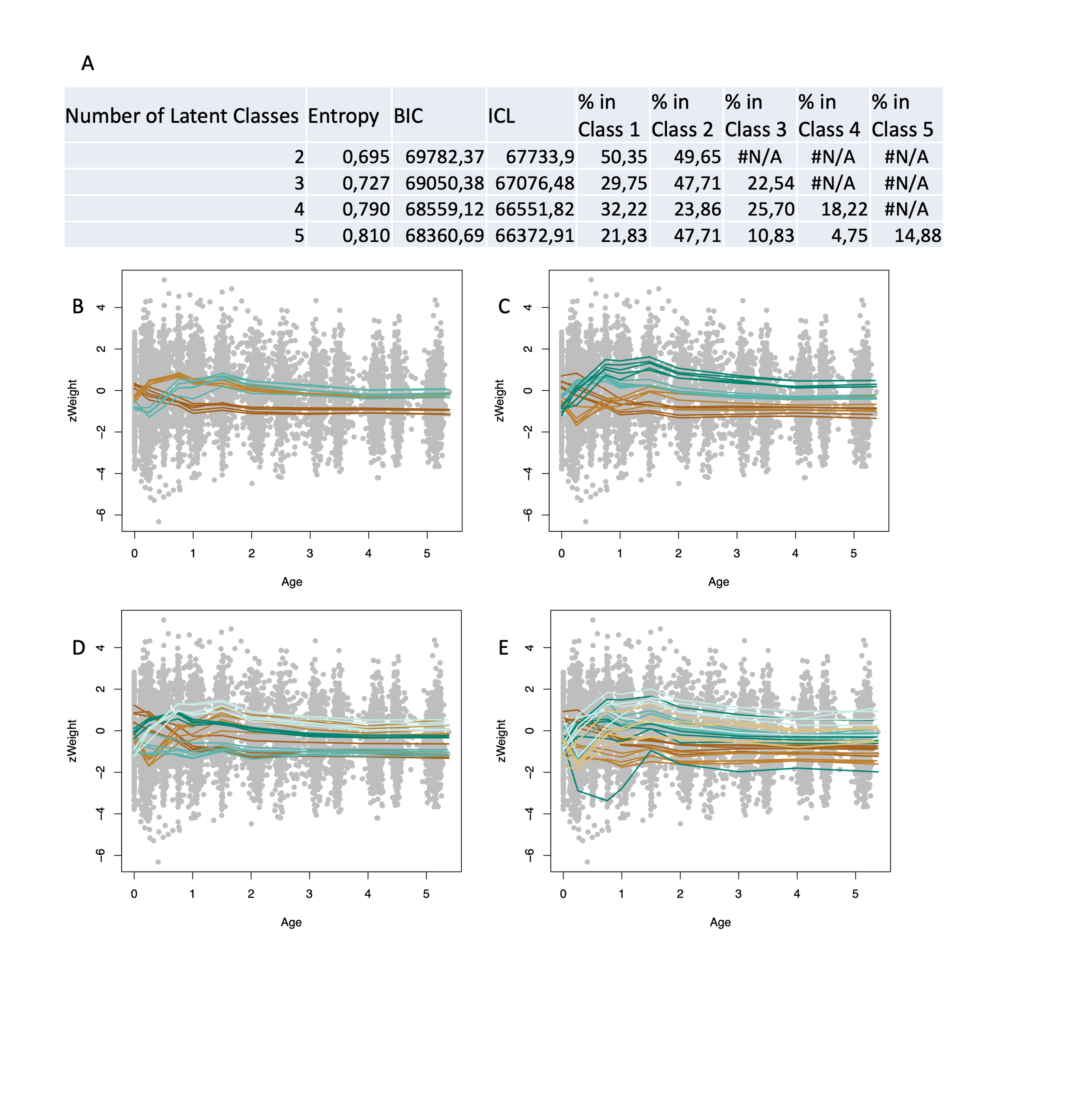

Supplement: S17 Fig — Information used to choose the appropriate value for k, the number of latent classes within zHeight + zWeight, given a piecewise linear spline model specification with knots places at (0.25,0.75,1,1.5,2,3,4). A) Fit statistics for k = (1:6). Profiles of LCMM Classes identified within zHeight + zWeight using a randomly selected 50% of subjects, repeated 10 times for B) k = 3, C) k = 4, D) k = 5 and E) k = 6, illustrated using zWeight. (TIF) [file pone.0319237.s019.tif]

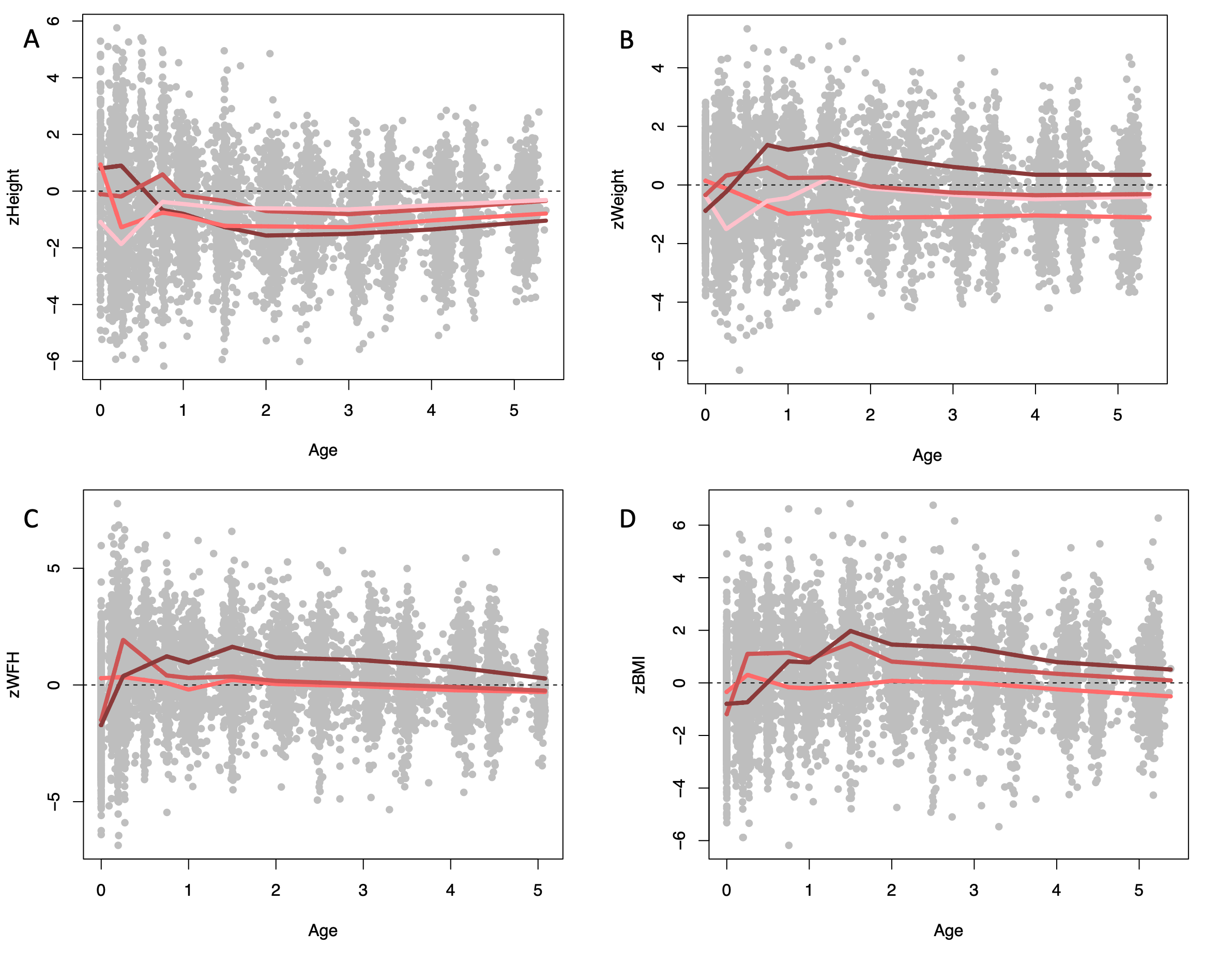

Supplement: S18 Fig — Latent Growth Trajectories identified within A) zHeight, B) zWeight, C) zWFH and D) BMI given additional knots placed at timepoints (0.25,0.75,1,1.5,2,3,4). (TIF) [file pone.0319237.s020.tif]

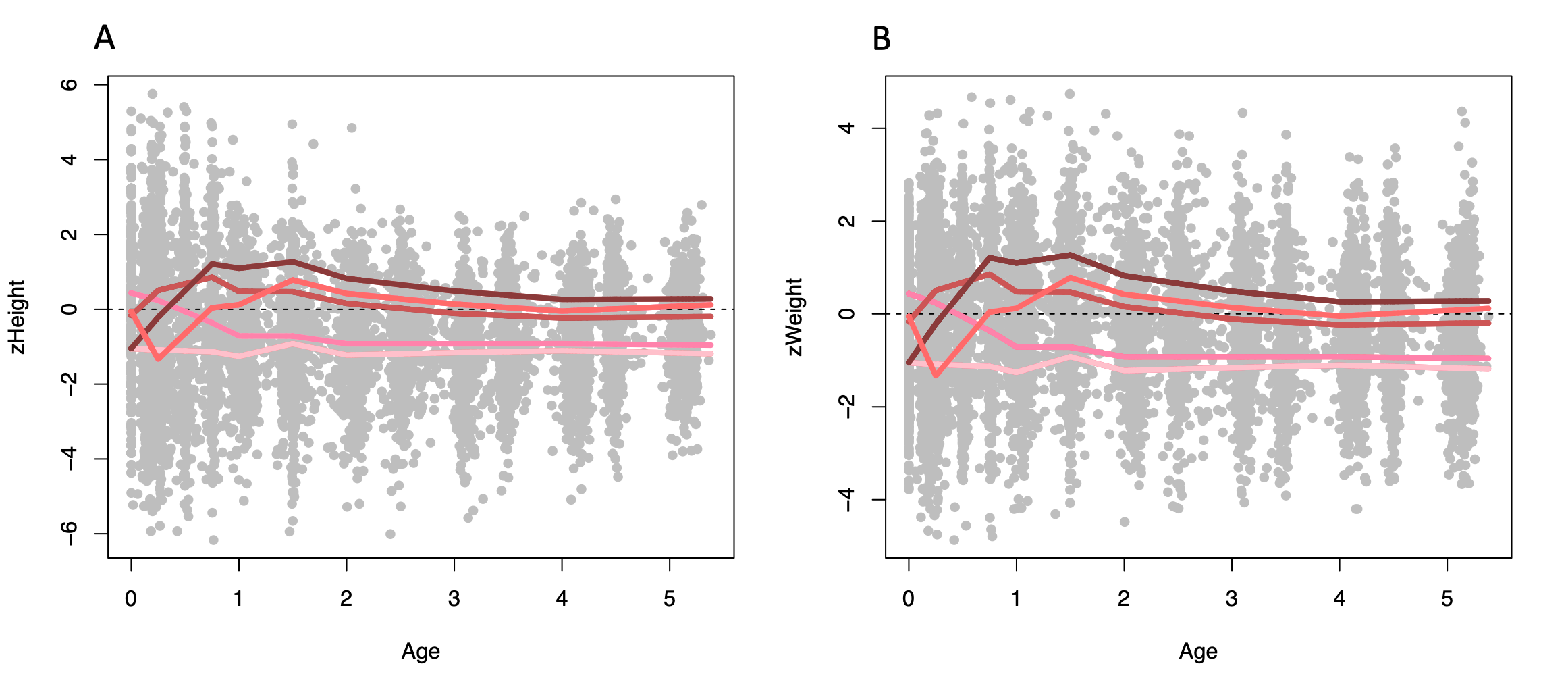

Supplement: S19 Fig — Latent Growth Trajectories identified within A) zHeight and B) zWeight as identified using the multivariate response of zHeight + zWeight, given additional knots placed at timepoints (0.25,0.75,1,1.5,2,3,4). (TIF) [file pone.0319237.s021.tif]

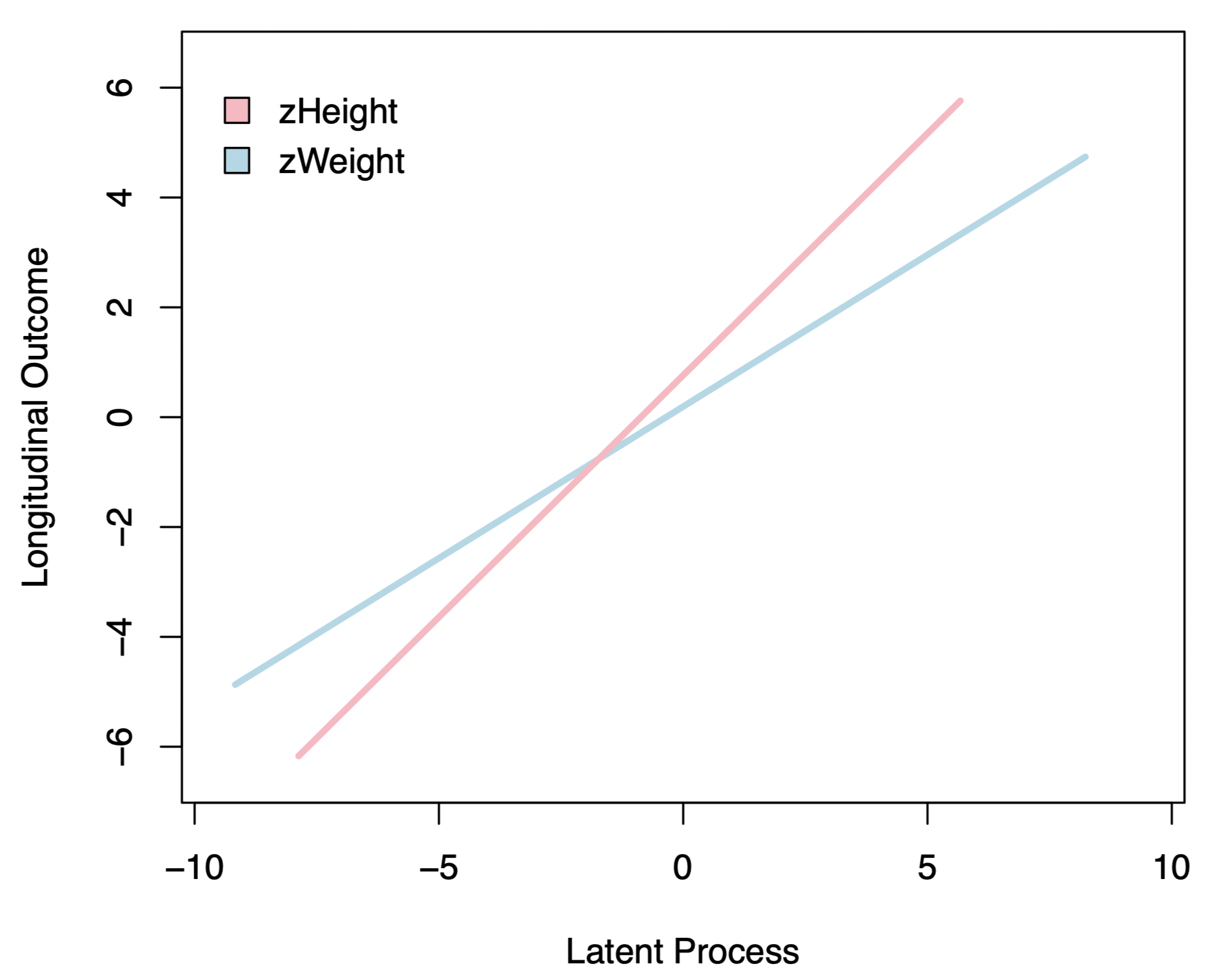

Supplement: S20 Fig — Linear link functions illustrating the relationship between zHeight and zWeight and the Latent Process within the multivariate zHeight + zWeight model. (TIF) [file pone.0319237.s022.tif]

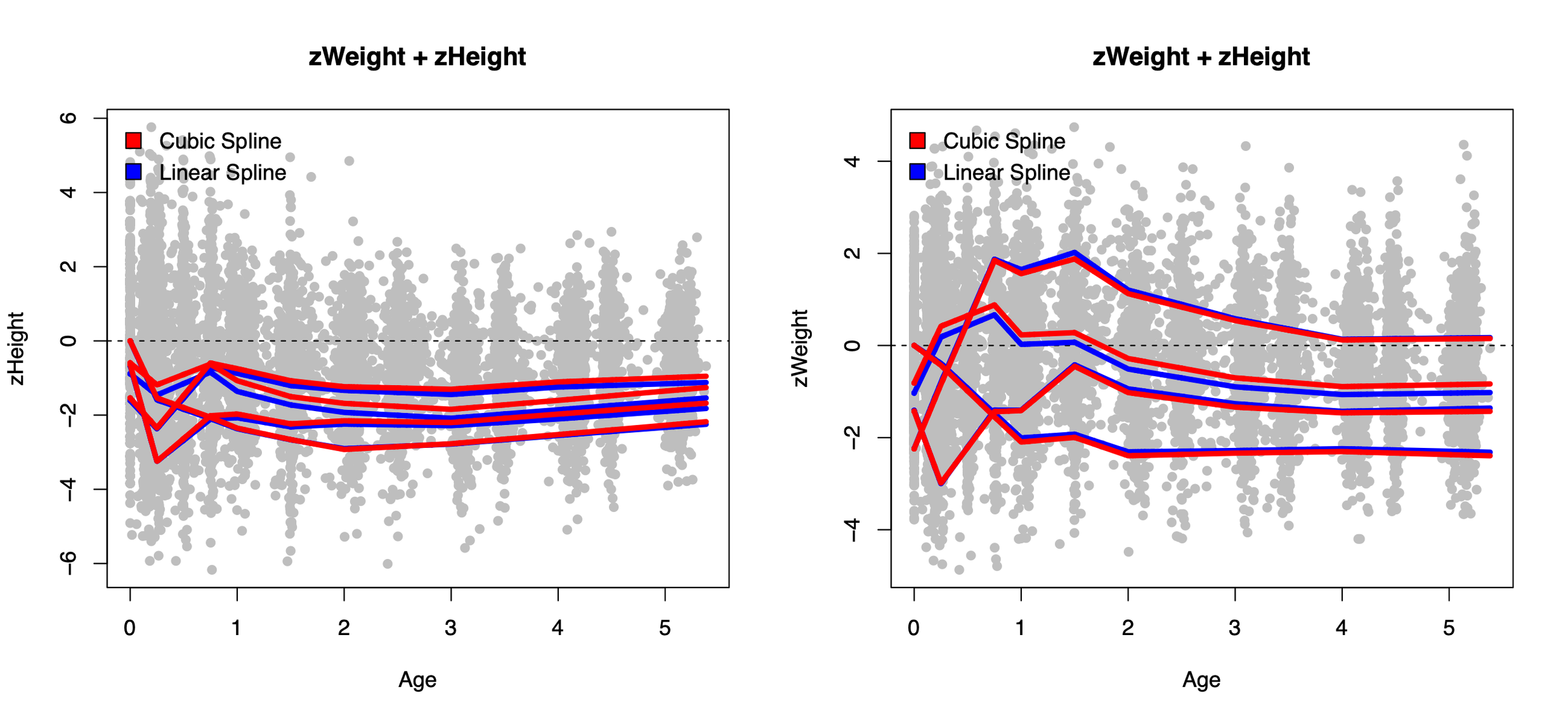

Supplement: S21 Fig — Trajectories of zHeight and zWeight profiles identified given four latent classes when considering a linear or cubic (with three equally spaced knots) spline link function specifying the relationship between the longitudinal outcomes and latent process. Here the effect of link is shown on k = 4; when k = 5 is considered the cubic spline approach identifies a class of outliers (n = 33) which does not meet the criteria for adequate class size, thus leading to k = 4 as the optimal number of classes considered. (TIF) [file pone.0319237.s023.tif]
