# Supplementary material for: Latent classes of anthropometric growth in early childhood using uni- and multivariate approaches in a South African birth cohort
Source: PLoS One. 2025 Mar 25;20(3):e0319237. doi: 10.1371/journal.pone.0319237 (PMC11936193; doi:10.1371/journal.pone.0319237)
Supplement: Attachment — Submitted filename: Response to Reviewers.docx [file pone.0319237.s040.docx]

Response to Reviewers

PONE-D-23-38575

27 November 2024

Latent Classes of Anthropometric Growth in Early Childhood Using Uni- and Multivariate approaches in a South African Birth Cohort

This question was raised within the introductory section of the response to the paper:

*We would like to inquire whether the researchers observed any relationships between rapid weight gain or lack of recovery and maternal BMI or other socioeconomic factors. In Kenya, for example, months of famine occur from February to May, which may intensify challenges for those who do not recover or who experience height recovery. We are also interested in whether they have considered family height targets and head circumference in their analysis.*

Thank you for this question, as this is raised again in the reviewer comments section it has been addressed there. The comment on this is found within the Reviewer #2, comment #3 section.

Journal Requirements:

1. Style Requirements

The manuscript has been altered to meet PLOS ONE's style requirements. These changes have not been recorded in the revised manuscript.

2. Financial Disclosure

The funders had no role in study design, data collection and analysis, decision to publish, or preparation of the manuscript.

3. Data Availability

Data cannot be shared publicly because of ethical conditions with which study investigators are obliged to comply. Access to the project data is restricted to nominated investigators approved by the University of Cape Town Human Research Ethics Committee, as per the consent document. Interested, qualified researchers may request to access this data by contacting the Drakenstein Child Health Study (via [lesley.workman@uct.ac.za](mailto:lesley.workman@uct.ac.za)) to submit a formal data use request and ensure required ethical approval received prior to use.

4. Ethics Section

The ethics section has been moved to the methods section as requested.

5. Supporting Information Files

Supporting Information files have been captioned at the end of the manuscript.

6. Review Reference List

The reference list has been reviewed and updated to ensure it is complete.

Reviewers Comments:

Reviewer #1:

| **Reviewer Comment** | **Response to Comment** | **Included within Manuscript** |
| --- | --- | --- |
| 1. In abstract (results), “…strong association was found between various growth classes and abnormal growth features….” The word “strong” need to be explained with values in the abstract. | *Thank you for this comment. To clarify this, the following was added to the abstract:* | *Much greater or lesser than expected proportions (an increase by 25% in some cases), when compared to the proportion of abnormal growth features across the entire cohort.* |
| 2. In the manuscript, the words of “Height” and “height,” and “Weight” and “weight” were mixed. They need to be uniformed. | *Thank you for this observation. Within the manuscript all “Height” have been changed to “height”, and all “Weight” have been changed to “weight”.* |  |
| 3. In methods, it is stated that “Pregnant women in a poor peri-urban area in South Africa, were enrolled during their second trimester of pregnancy, from March 2012 – March 2015.” It should be explained more on the study population and selection procedures, e.g., target community population, expected annual number of pregnancies, number of identified pregnancies by the study, selection criteria, urine pregnancy test done by the study. | *Thank you for this comment, to elaborate details with respect to recruitment we have prepared the following:*  *Mothers were recruited at an antenatal clinic, and hence pregnancy was already established by the primary healthcare providers. Therefore, no pregnancy test were performed by study staff. Mothers were enrolled at 20-28 weeks as this is when the antenatal scans are performed.* | *The following was added to the methods section:*  *Mothers were recruited at an antenatal clinic, and all who were over the age of 18 and intended to reside in the Paarl area for at least one year were eligible for this study.* |
| 4. The standardized anthropometric values depend on the accuracy of gestational age at birth. How this study assured the accuracy of gestational age at birth. This might be explained in a previous publication of this cohort, but this point is critical for this modelling study, it is important to explain it, e.g., the last menstruation period is based on mother’s recall, or the study used ultrasound-based gestational age at enrolment? | *Thank you for this important comment, to respond to this the following was added to the methods section:* | *Gestational age was calculated using second trimester ultrasound. In the event that this was unavailable, last menstrual period was used to calculate gestational age at the time of enrolment.* |
| 5. Page 6, Line 2, “Measures within 0.5cm and 0.1kgs…” should be corrected as “Measures within 0.5cm and 0.1kg….” | *This change has been made.* |  |
| 6. It might be more interesting for international readers if other ethnicities studies can be discussed on the early childhood growth patterns. As this study demonstrated, there has been studies reporting that sub-Saharan African children were mostly born in normal size, then deteriorate towards 2-3 years of age. On the other hand, South Asian children tend to be born small and track various pattern during the childhood. It is important to discuss these patterns can be discussed with the internal (biological) and external (environmental) factors. | *Thank you for this point, to comment on this we have included the following to the discussion:* | *Stein et al., (2010) compared the patterns of height in early childhood (birth to age 5) in five birth cohorts from distinct populations around the globe (Brazil, Guatemala, India, Philippines and South Africa) (18). Considering the same cohorts, Poveda Rey et al., (2021) looked at weight throughout early childhood (19).* These studies illustrated diversity in growth profiles across multiple LMIC nations.  *The Brazilian cohort mostly followed expected standardised height and expected standardised weight within the first five years of life. While the Guatemalan, South African and Indian cohorts were born smaller (with respect to length) than the expected; the South African cohort recovered by the age of five while the Guatemalan and Indian cohorts did not. The Guatemalan, Filipino and South African cohorts were born weighing slightly less than expected. The South African cohort did not recover, nor did they show further reduction in expected weight. However, the Guatemalan and Filipino cohorts did show further reduction in standardised weight scores, to a greater extent for the Filipino cohort. The Filipino cohort was born with an expected height but decelerated to much smaller than expected by one year of age. The Indian cohort was born with the smallest standardised weight which did not improve through early childhood. Interestingly, all cohorts showed a decreased standardised height at two years compared to birth; while some were able to recover from this deceleration (Brazil, South Africa), other cohorts did not (Guatemala, India, Philippines).* |
| 7. There is no discussion on the mode of delivery. This relates to distinguished characteristics of newborn gut environment as reported in many publications. Moreover, caesarean section has been rapidly increased in LMIC. | *Thank you for this comment. We have added the following to the methods section:* | *The most common mode of delivery was natural birth, while 20.12% of the children within the study were delivered by caesarean-section.* |

Reviewer #2:

| **Reviewer Comment** | **Response to Comment** | **Included within Manuscript** |
| --- | --- | --- |
| 1. The ideal timing for obtaining measurements. | *To explain this in detail, we have prepared the following, however this has not been added into the manuscript:*  *After birth, there is an initial period of accelerated growth which then slows down by one year of age. Considering weight, children double their birth weight around 5-6 months of age and triple their weight it at one year of age. Between 1-2 years children then gain 2.5 kg on average. Considering height, from birth to 6 months infants grow on average 2.5 cm a month which slows to 1.25 cm a month from 7 to 12 months and then to 10 to 12 cm between 1 and 2 years.* | *To clarify this, the following was added into the methods section:*  *The most rapid period of growth through childhood is during infancy, specifically throughout the first 2 years, hence measurements were captured more frequently through this period.* |
| 2. The cutoff point for identifying growth patterns. The study employs a method similar to that used for English children, utilizing 9 centiles, which corresponds to 0.67 SD between each centile. This approach appears to be more effective than the WHO standards, which classify overweight as weight for height greater than 2 SD and obesity as greater than 3 SD. | *Here there may be a misunderstanding of our approach used, to clarify this we respond with:*  *To prepare the observed height, weight and BMI data for the longitudinal modelling and latent class analysis process the WHO approach of standardisation was used. This approach compares observed data to expected height/ weight/ bmi reference ranges given child age and gender to allocate a zScore. This zScore is close to 0 if a child has a growth measurement close to the expected value for their specific age and gender, or a larger positive or negative value if the child is smaller or larger than expected respectively. Once these zScores are calculated no cut-offs are employed within the longitudinal modelling and latent class identification process. If the reviewer perhaps meant to question the cutoff used with respect to RWG, as mentioned in the methods, RWG during the first nine months was identified. However, when identifying the proportion of children belonging to abnormal growth profiles (stunted, obese or overweight) within the latent growth profiles then the WHO standards were indeed used.* |  |
| 3. Additionally, we would like to inquire whether the researchers observed any relationships between rapid weight gain or lack of recovery and maternal BMI or other socioeconomic factors. In Kenya, for example, months of famine occur from February to May, which may intensify challenges for those who do not recover or who experience height recovery. We are also interested in whether they have considered family height targets and head circumference in their analysis. | *To answer this briefly, yes. However, this is not in the scope of this analysis. We are currently working on a separate paper that explores the relationship between latent growth profiles and determinants of such growth which includes socioeconomic factors and measures of maternal BMI as well as many other variables. We chose to focus on these as separate analyses to allow a thorough and detailed exploration of both class identification and predictor analysis. We have not looked at family height targets and head circumference as these measures still require a large amount of data cleaning before they ready to be used, and once again this is not in the scope of this analysis as we have focused on Height, Weight and composite measures of these two variables.* |  |
